# Overexpression of DDIT4 and TPTEP1 are associated with metastasis and advanced stages in colorectal cancer patients: a study utilizing bioinformatics prediction and experimental validation

**DOI:** 10.1186/s12935-021-02002-x

**Published:** 2021-06-09

**Authors:** Fahimeh Fattahi, Jafar Kiani, Mahdi Alemrajabi, Ahmadreza Soroush, Marzieh Naseri, Mohammad Najafi, Zahra Madjd

**Affiliations:** 1 Oncopathology Research Center, Iran University of Medical Sciences, (IUMS), Tehran, Iran; 2 Department of Molecular Medicine, Faculty of Advanced Technologies in Medicine, Iran University of Medical Sciences, Tehran, Iran; 3 Firoozgar Clinical Research Development Center (FCRDC), Iran University of Medical Sciences, Tehran, Iran; 4 Obesity and Eating Habits Research Center, Endocrinology and Metabolism Clinical Sciences Institute, Tehran University of Medical Sciences, Tehran, Iran; 5 Biochemistry Department, Faculty of Medical Sciences, Iran University of Medical Sciences, Tehran, Iran

**Keywords:** Colorectal cancer (CRC), Bioinformatics analysis, DDIT4, TPTEP1

## Abstract

**Background:**

Various diagnostic and prognostic tools exist in colorectal cancer (CRC) due to multiple genetic and epigenetic alterations causing the disease. Today, the expression of RNAs is being used as prognostic markers for cancer.

**Methods:**

In the current study, various dysregulated RNAs in CRC were identified via bioinformatics prediction. Expression of several of these RNAs were measured by RT-qPCR in 48 tissues from CRC patients as well as in colorectal cancer stem cell-enriched spheroids derived from the HT-29 cell line. The relationships between the expression levels of these RNAs and clinicopathological features were analyzed.

**Results:**

Our bioinformatics analysis determined 11 key mRNAs, 9 hub miRNAs, and 18 lncRNAs which among them 2 coding RNA genes including DDIT4 and SULF1 as well as 3 non-coding RNA genes including TPTEP1, miR-181d-5p, and miR-148b-3p were selected for the further investigations. Expression of DDIT4, TPTEP1, and miR-181d-5p showed significantly increased levels while SULF1 and miR-148b-3p showed decreased levels in CRC tissues compared to the adjacent normal tissues. Positive relationships between DDIT4, SULF1, and TPTEP1 expression and metastasis and advanced stages of CRC were observed. Additionally, our results showed significant correlations between expression of TPTEP1 with DDIT4 and SULF1.

**Conclusions:**

Our findings demonstrated increased expression levels of DDIT4 and TPTEP1 in CRC were associated with more aggressive tumor behavior and more advanced stages of the disease. The positive correlations between TPTEP1 as non-coding RNA and both DDIT4 and SULF1 suggest a regulatory effect of TPTEP1 on these genes.

**Supplementary Information:**

The online version contains supplementary material available at 10.1186/s12935-021-02002-x.

## Background

Colorectal cancer (CRC) is the second most common cancer and leading cause of cancer-related deaths in the world [1]. CRC is now known to be a heterogeneous disease due to the various genetic and epigenetic alterations causing the disease [2]. The existence of a subset of cancer cells named cancer stem cells (CSCs) also leads to tumor heterogeneity by utilizing self-renewal and multi-lineage differentiation features in the tumor [3]. These alterations and CSCs play important roles in development and progression of CRC [4, 5]. CRC is typically classified according to the pathological and clinical features of the American Joint Committee on Cancer (AJCC) and the staging system is used to evaluate prognosis and guide treatment strategies [6, 7]. There are some genetic biomarkers which can aid in estimating prognosis and in guiding treatment selection in CRC patients such as 18q loss of heterozygosity (LOH), p27 Kip1, DNA microsatellite instability [7], K-RAS mutation [8] and RNA expression profile [9–11]. It is important to find sensitive and specific biomarkers to best guide early and appropriate treatment before disease progression [12].

Bioinformatics can serve as a very useful tool to investigate the complexity of big datasets, discover novel biomarkers and analyze their validation in clinical studies [13].

Nowadays, some RNA expression panels are used in clinical cancer such as PAM50 [14]. Although the main focus is on transcripts of coding RNA genes, there are some evidence that non-coding RNAs (ncRNAs) are also involved in hallmarks and pathological processes of cancer [15, 16]. The recent discovery from whole genomes sequencing has revealed that 98% of the human transcriptome contain ncRNAs [17]. Evidence shows that the biological functions of many ncRNAs that are involved in the diseases are unknown. The biology of microRNAs (miRNAs) as the abundant small ncRNAs has been better understood [18, 19]. They can interfere in tumorigenesis by regulating oncogenes and tumor suppressor genes [20]. Small ncRNAs that regulate mRNAs can be predicted by numerous in-silico computational programs [21, 22]. Long non-coding RNAs (lncRNAs) are another type of ncRNAs which are expressed in tissue-specific pattern and dysregulated in cancer [23] and play important functions in cellular processes such as cell proliferation, motility, and apoptosis [24]. Some reports have demonstrated that the levels of some lncRNAs, miRNAs and mRNAs are controlled and regulated by each other in cancer [25]. Identification of the interacting target RNAs of each lncRNA is an important step in understanding lncRNA functions which can be done through computational prediction of lncRNA–RNA interactions [26]. In the current study, by getting help from bioinformatics analysis and computational algorithms, we selected several genes for further investigation of RNA levels in our CRC patients. These genes included DNA-damage-inducible transcript 4 (DDIT4), sulfatase 1 (SULF1) as coding RNA genes and miR-181d-5p, miR-148b-3p and TPTE Pseudogene 1 (TPTEP1) as ncRNA genes.

DDIT4 also known as REDD1 or RTP801, is expressed in response to diverse stress conditions and its abnormal expression is linked to cancer via the effects on PI3K/Akt/mTOR signaling [27, 28]. In-Silico evaluation has shown dysregulation in RNA expression levels of DDIT4 in several cancers which may be used as a poor prognostic factor in colon cancer [29].

SULF1 is a sulfatase that selectively remove 6-O-sulfate groups from heparan sulfate (HS). Alternation of HS chains is important in signaling events because heparan sulfate proteoglycans (HSPGs) are released into the extracellular matrix and act as co-receptors which contributes to regulation of cellular processes [30]. Some studies have reported dysregulation of SULF1 expression in CRC [31–33].

It was described in a meta-analysis report that dysregulation of miRNA-181d family membrane can be used as prognostic marker in different cancers [34]. Also, dysregulation of miRNA-148b-3p expression was reported in numerous cancers including breast [35], thyroid [36], prostate [37], colorectal [38] and gastric cancer [39]. Until now, dysregulated expression of TPTEP1 has been reported mainly in patients with human lung [40] and liver cancer [41].

In this study, we explored effector networks of mRNAs, miRNAs, and lncRNAs in CRC based on predicted relationships of these RNAs via bioinformatics tools. DDIT4, SULF1, miR-181d-5p, miR-148b-3p and TPTEP1 were selected as the potential biomarkers in CRC patients and their expression levels were measured by RT-qPCR. Also, we investigated the association between these RNA expression levels and clinicopathological features in CRC tissue samples. Although some of these RNAs were reviewed in CRC in the past, there is no data about RNA expression levels of DDIT4 and TPTEP1 and their clinical significance in CRC patients as well as in the colorectal CSC-enriched spheroids. Therefore, based on our knowledge, our study is the first to report these data, and also to explore the correlations of these RNA expression levels amongst each based on our prediction analysis via bioinformatics tools.

## Methods

### Bioinformatics prediction study

#### Data sources and network construction

**Fig. 1:**
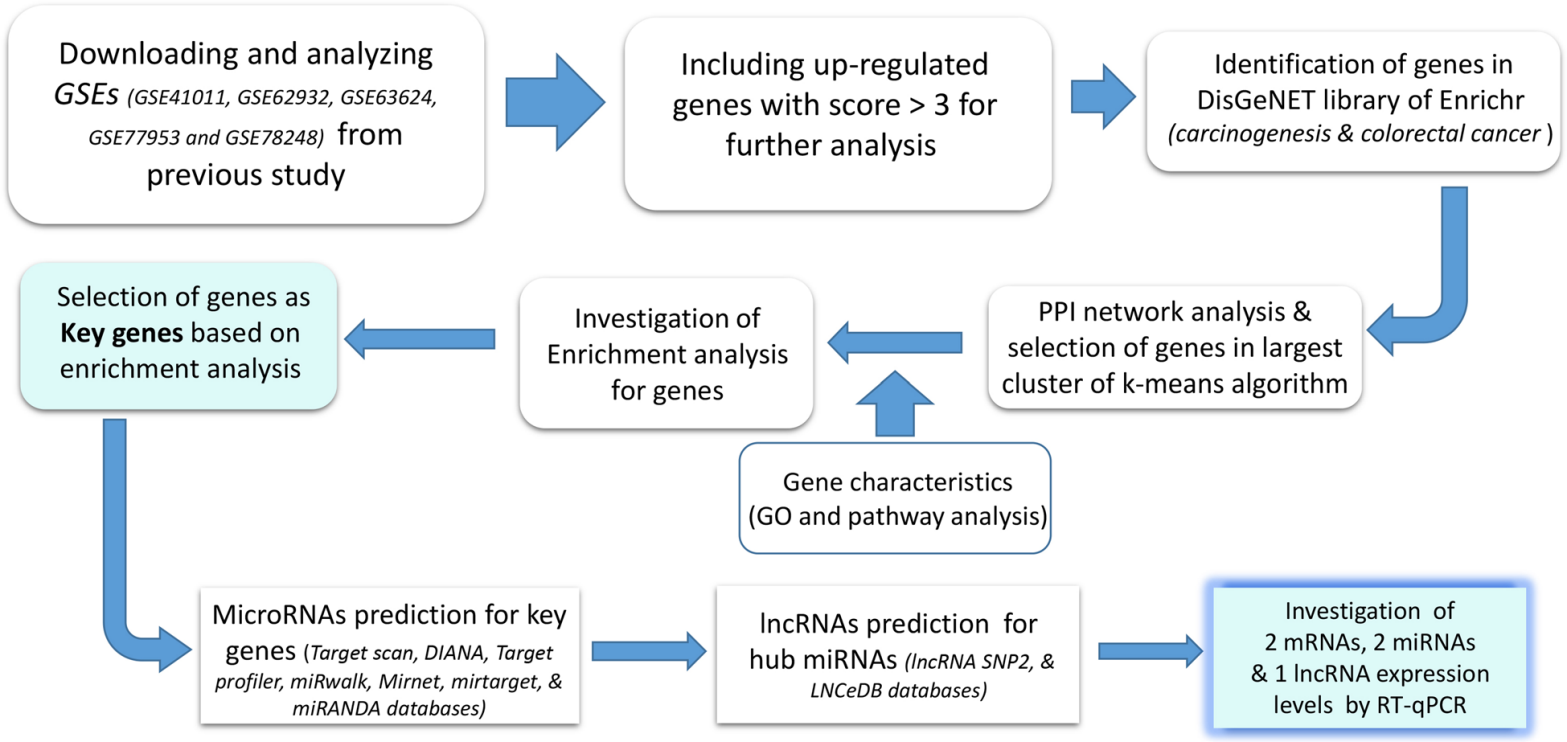
Bioinformatics analysis workflow. This figure summarizes the steps and tools in order to select genes for our experimental work

In our previous study, we detected differentially expressed genes (DEGs) in total 231 CRC patients obtained from merged five data series on Gene Expression Omnibus (GEO), including GSE41011, GSE62932, GSE63624, GSE77953, and GSE78248. Up-regulated genes with score > 3 were included in the current study, the mean score of up-regulated genes was used as cut-off criteria (Additional file 1: Table S1). The score was obtained from the merging series mentioned above as we described previously [42]. In the following step, the genes which were highly associated with carcinogenesis and colorectal cancer diseases (*P* < *0.0001*) were screened among the up-regulated genes according to the DisGeNET library [43] on Enrichr [44]. Protein–protein interaction (PPI) network was found using STRING database with stringApp (confidence score > 0.4) [45] in Cytoscape software [46]. K-means algorithm was used for clustering of the STRING database, and the genes in the largest cluster were selected as the entry criteria for subsequent analysis. Workflow of bioinformatics analysis steps were descripted in Fig. 1.

#### Network enrichment analysis

The pathway enrichment analysis and Gene Ontology (GO) were done using Enrichr in order to better understanding the biological process and functions of genes. Enrichr is a powerful enrichment analysis online tool which is linked to mammalian gene sets libraries and pathway databases [44]. We used KEGG [47], Reactome [48], BioPlanet [49], and WikiPathways [50] which are important databases and store a lot of data on biological pathways, for our pathway analysis on Enrichr. The key genes for the present study were selected according to pathway and GO analysis. To visualize results of pathway and GO analysis for key genes, ClueGO plug-in by Cytoscape software was used [51].

#### mRNA–miRNA network and lncRNA predictions

Prediction of MicroRNAs was performed for key genes using Target scan [52], DIANA [53], miR2Disease [54], miRWalk [55], miRNet [56], and microRNA.org [57]*.* mRNA**–**miRNA bipartite network was constructed for experimented and high-quality predicted mRNA**–**miRNA data. The highest degree and most common miRNAs for key genes were selected as hub miRNAs of mRNA**–**miRNA bipartite network. Finally, in order to investigate the communication of the miRNAs with other RNAs, lncRNA predictions were performed through the hub miRNAs on the lncRNA SNP2 [58], miRwalk [55], LNCeDB [59] databases.

#### Selecting genes process among the key genes for experimental study

Enrichment analysis and literature review led to selecting two mRNAs and miRNAs among numerous key genes and hub miRNAs related to CSCs for our experimental study. Also, we selected one of topmost lncRNAs that has putative target site interaction found via computational prediction of lncRNA**–**mRNA on http://rtools.cbrc.jp/cgi-bin/RNARNA/index.pl [60] based on RactIP [61] and IntaRNA databases [62].

### Experimental studies in CRC tissues and colorectal CSC-enriched spheroids

#### Tissue specimens and clinical data collection

Forty-eight fresh tissue samples (tumor and adjacent normal tissues as control) from the patients with CRC, who have not received any preoperative radiotherapy and other antitumor therapy, were harvested through the surgery at the Firoozgar and Bahman hospitals (Tehran, Iran) between April 2017 and May 2018. All samples were transferred to RNA later (EURx, Poland) immediately after resection and placed into prepared cryogenic vials, and frozen in liquid nitrogen to avoid RNA degradation. The diagnosis of CRC was made by postoperative pathological examination according to the diagnostic criteria from the AJCC [7]. Clinicopathological data from the patients were collected from their electronic medical record system. Clinicopathological features for tumor included: tumor size, vascular invasion, perineural invasion, TNM stage, metastasis, and histologic grade (tumor differentiation) in addition to the sex and age of the patients.

#### Isolation and confirmation of colorectal CSC-enriched spheroids

Colorectal CSC-enriched spheroids were isolated from the HT-29 cancer cell line which were purchased from Iranian Biological Resource Center (IBRC; C10097, RRID: CVCL_0320). The HT-29 cancer cells were cultured in DMEM/High glucose medium (Gibco, Germany) supplemented with 10% fetal bovine serum (Gibco, Germany), 1% non‐essential amino acids (Gibco, Germany), 2 mM l-glutamine (Gibco, Germany) and 1% Penicillin–Streptomycin (Biowest, France) at 37 °C in 5% CO_2_ and 95% humidified incubator. Colorectal CSC-enriched spheroids were generated using hanging droplet technique of HT-29 cancer cells descripted earlier [63]. Spheroid culture medium included DMEM/F12 (Gibco, Germany), supplemented with 2% B27 (Gibco, Germany), 10 ng/ml of basic fibroblast growth factor (bFGF), 20 ng/ml epidermal growth factor (EGF) (PeproTech, USA), 1% nonessential amino acids, 2 mM l-glutamine, and 1% Penicillin–Streptomycin. In brief, 70%‐80% confluent HT‐29 cells were detached with 0.05% trypsin/EDTA (Gibco, Germany) and were washed twice with PBS and serum‐free medium. Then, 25 μL droplets containing 10,000 cells re-suspended in spheroid culture medium on the lid of Petri dishes containing 5 ml PBS at 37 °C incubator with 5% CO_2_ and 95% humidified incubator for 96 h. Drops containing spheroids were harvested by washing with a gentle shaking of media and transferred onto nonattachment flasks (coated flasks with 1.2% poly-HEMA (Sigma, USA)) for 6 days in spheroid culture medium conditions as described above. After observation of sphere morphology by microscope, spheroids were evaluated for CSC features based on RNA expression of stemness genes (OCT4, SOX2, C-MYC, KLF4 and NANOG), ABC transporter genes (ABCB1, ABCG2, and ABCC1) and epithelial-mesenchymal transition (EMT) genes (TWIST1, SNAIL1, Vimentin, and ZEB1) [64].

#### RNA extraction and cDNA synthesis

Total RNA was extracted from frozen tissues and cells (HT-29 cell line and colorectal CSC-enriched spheroids), using miRNeasy mini kit (QIAGEN GmbH-Germany) according to the manufacturer’s instructions. RNA samples were separated by agarose gel electrophoresis and their concentration was measured by optical absorbance at 260/280 nm. Complementary DNA (cDNA) was synthesized from extracted RNA using cDNA synthesis kit (TaKaRa Bio, Shiga, Japan) and miRNA cDNA synthesis kit (Bon Yakhteh, Iran).

#### Real time-quantitative polymerase chain reaction (RT‐qPCR)

The specific primers for amplification with RT-qPCR were designed using Primer-BLAST [65] and OligoAnalyzer 3.1 software (Integrated DNA Technologies) (Table 1). RT-qPCR was performed to find the expression levels of selected genes from bioinformatics analysis and stemness, ABC transporter and EMT genes that were used for validating colorectal CSC-enriched spheroids. RT-qPCR reactions were performed by SYBR Green PCR Master Mix (Takara, Japan) on Real-Time PCR System (Rotor-Gene Q MDx, Germany). The expression of miRNA genes and TPTEP1 was normalized to internal control of kit and RNU6 (U6) expression levels, respectively. To normalize other mRNAs expression, GAPDH gene was used as an internal control gene. The relative expression levels of the genes were calculated by 2^−ΔΔCt^ method [66].

**Table 1:** Primer sequence of genes for RT-qPCR

Gene groups	Gene names	Primer Sequence (5´ → 3´)
Selected genes from bioinformatics analysis	DDIT4	F: CTTTGGGACCGCTTCTCGTC R: GGTAAGCCGTGTCTTCCTCCG
	SULF1	F: GGACGGATACAGCAGGAACG R: CAGCACATGGGTGTAGTCACA
	TPTEP1	F: AGCCGCAGACAAAAGACCTCGG R: CCACCAAACAGGCTTCGTGTGA
	miRNA-181d-5p	AACATTCATTGTTGTCGGTGGGT
	miRNA-148b-3p	TCAGTGCATCACAGAACTTTGT
Stemness genes	OCT4	F: GTGGAGAGCAACTCCGATG R: TGCAGAGCTTTGATGTCCTG
	SOX2	F: AATGGGAGGGGTGCAAAAGAGG R: GTGAGTGTGGATGGGATTGGTG
	C-MYC	F: ACACATCAGCACAACTACG R: CGCCTCTTGACATTCTCC
	KLF4	F: CCTCGCCTTACACATGAAGAG R: CATCGGGAAGACAGTGTGAAA
	NANOG	F: AGCTACAAACAGGTGAAGAC R: GGTGGTAGGAAGAGTAAAGG
EMT genes	TWIST1	F: TTCTCGGTCTGGAGGATGGAG R: ACGCCCTGTTTCTTTGAATTTGG
	SNAIL1	F: CCAGAGTTTACCTTCCAGCA R: GATGAGCATTGGCAGCGA
	VIM	F: TCTACGAGGAGGAGATGCGG R: GGTCAAGACGTGCCAGAGAC
	ZEB1	F: CTTCTCACACTCTGGGTCTTATTC R: CGTTCTTCCGCTTCTCTCTTAC
ABC transporter genes	ABCB1	F: GTTCAGGTGGCTCTGGATAAG R: AGCGATGACGTCAGCATTAC
	ABCG2	F: TTCCACGATATGGATTTACGG R: GTTTCCTGTTGCATTGAGTCC
	ABCC1	F: CGCCTTCGCTGAGTTCCT R: TGCGGTGCTGTTGTGGTG
Housekeeping genes	GAPDH	F: CATGAGAAGTATGACAACAGCCT R: AGTCCTTCCACGATACCAAAGT
	RNU6 (U6)	F: TCGCTTCGGCAGCACATATAC R: GCGTGTCATCCTTGAGCAG

#### Statistical analysis

Statistical analysis was performed using SPSS 21.0 software (SPSS Inc, Chicago, IL). All data in statistical analyses were expressed as median of RNA expression levels. Significance differences in expression levels of candidate genes between tumor and adjacent normal tissue samples as well as between colorectal CSC-enriched spheroids and HT-29 cell line were analyzed using nonparametric test (Mann–Whitney *U* test). For comparisons of quantitative values between more than two groups, Kruskal–Wallis test was used. The Spearman's test was applied to evaluate the association between expression levels of these RNAs amongst each other and clinicopathological features. P-value less than 0.05 was considered as statistically significant. GraphPad Prism version 8 software (GraphPad Software, La Jolla, CA) was used for making the boxplots, heat map graph and scatterplots.

## Results

### Bioinformatics analysis and selecting target genes

#### Network analysis and clustering genes

**Fig. 2:**
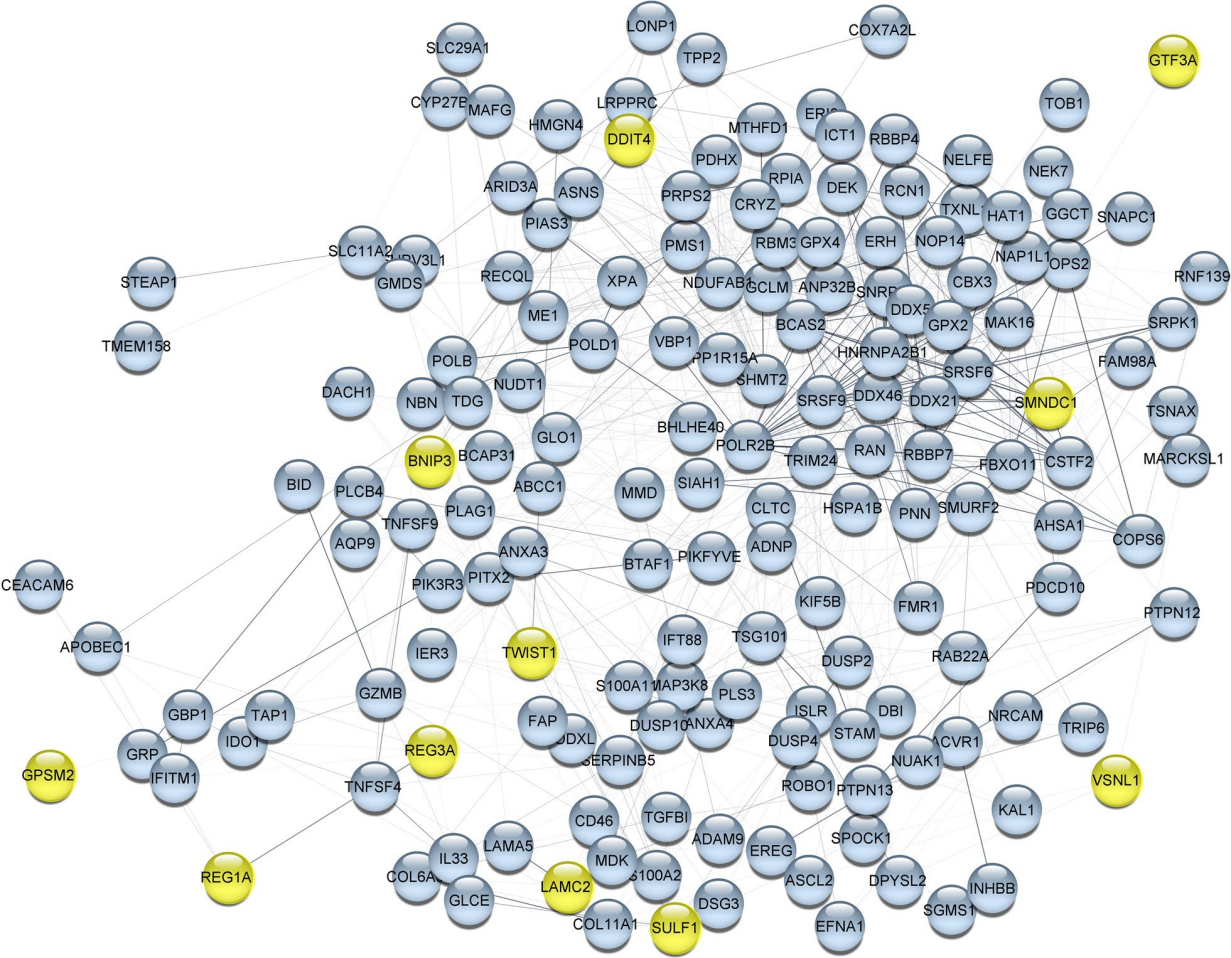
Protein–protein interaction network (PPI). PPI network analysis was done for the largest cluster of k-means based on stringApp (confidence score ≥ 0.4) in Cytoscape, yellow color nodes indicated key genes that were selected based on the enrichment analysis and literature review

Three hundred and seventy up-regulated genes were included in network analysis based on score > 3 which found to be involved in carcinogenesis or colorectal cancer (*P* < *0.0001*) on DisGeNET (Additional file 1: Table S1). PPI network analysis explored the interactions of these up-regulated genes amongst each other. Five main clusters were obtained from k-means algorithm for genes with confidence ≥ 0.4 (Additional file 2: Figure S1). In order to limit the number of genes, largest cluster covering 167 genes (Additional file 1: Table S1) were selected for subsequent analysis in which their PPI network is shown in Fig. 2.

#### Pathway and GO enrichment analysis

To find better characteristics of the 167 genes, pathway and GO enrichment analysis were performed using the Enrichr tool. The top 10 results of pathway and GO annotation analysis (*P* < *0.05*) were shown in the Additional file 2: Figure S2 and S3. Enrichment analysis displayed the “spliceosome”, “miRNA biogenesis”, “P53 signaling pathway”,” DNA repair”, “MAPK signaling pathway” and “gene expression” are parts of the top 10 pathways.

**Fig. 3:**
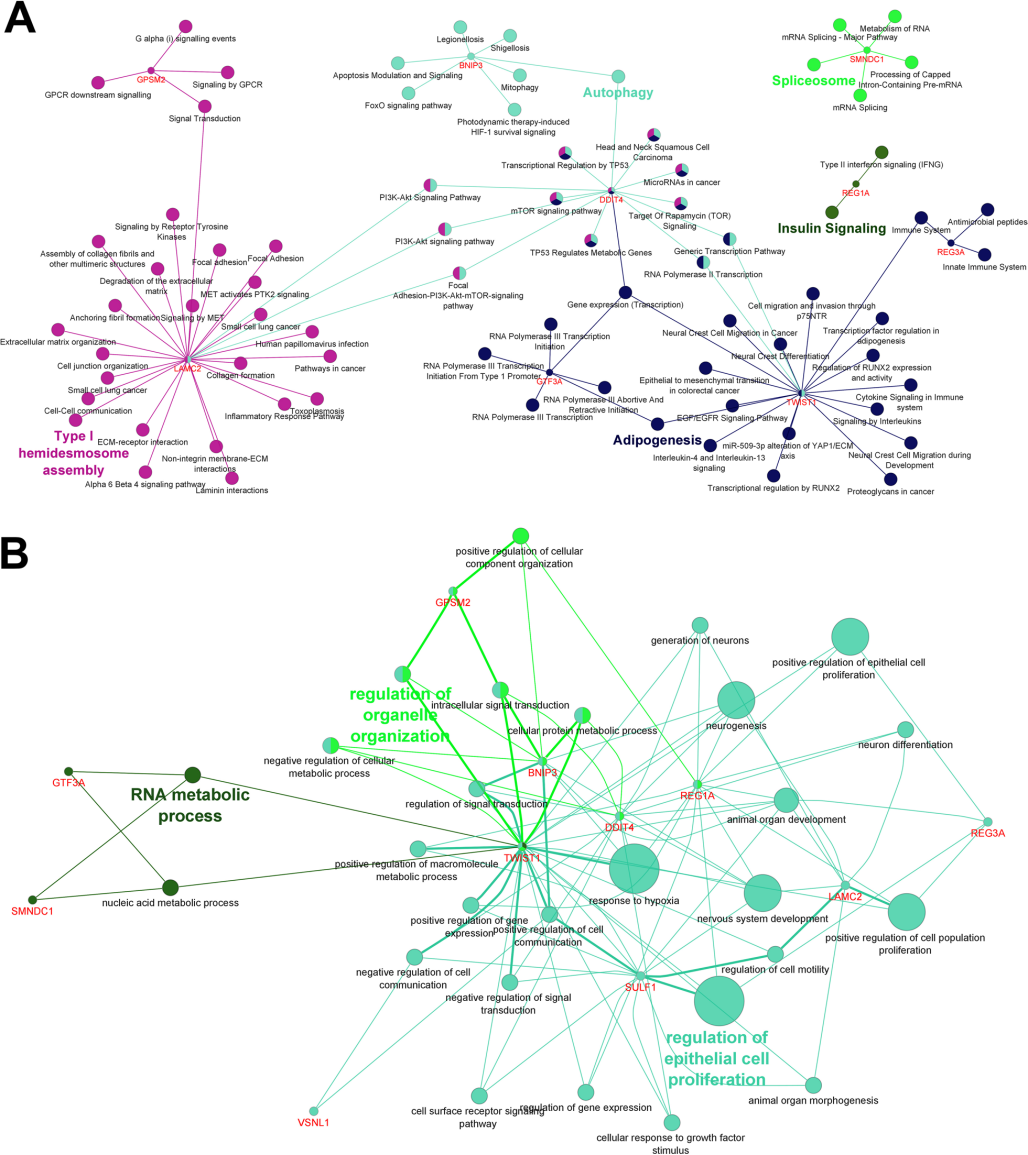
Pathway and gene ontology (GO) analysis of selected genes as key genes using the ClueGO plugin in Cytoscape. Pathway analysis, based on KEGG, Reactome, and Wikipathways (**A**), Common results of GO analysis (**B**) for 11 key genes

A closer check of GO and pathway analysis indicated that some of the genes participate in “microRNAs in cancer”, “proteoglycans in cancer”, “apoptosis” and “cell cycle” pathways. These genes contribute to several key biological processes including “extracellular matrix organization”, “regulation of cell migration” and “positive regulation of cell proliferation” based on GO analysis which their disorder was reported in cancer [67]. Information of functional characteristics of genes led to restricting these genes to 11 key genes (TWIST1, DDIT4, LAMC2, SULF1, REG1A, REG3A, VSNL1, BNIP3, GPSM2, GTF3A and SMNDC1). The information pathways and common features of 11 key genes are summarized in Fig. 3.

#### mRNA–miRNA network and prediction of lncRNAs

**Fig. 4:**
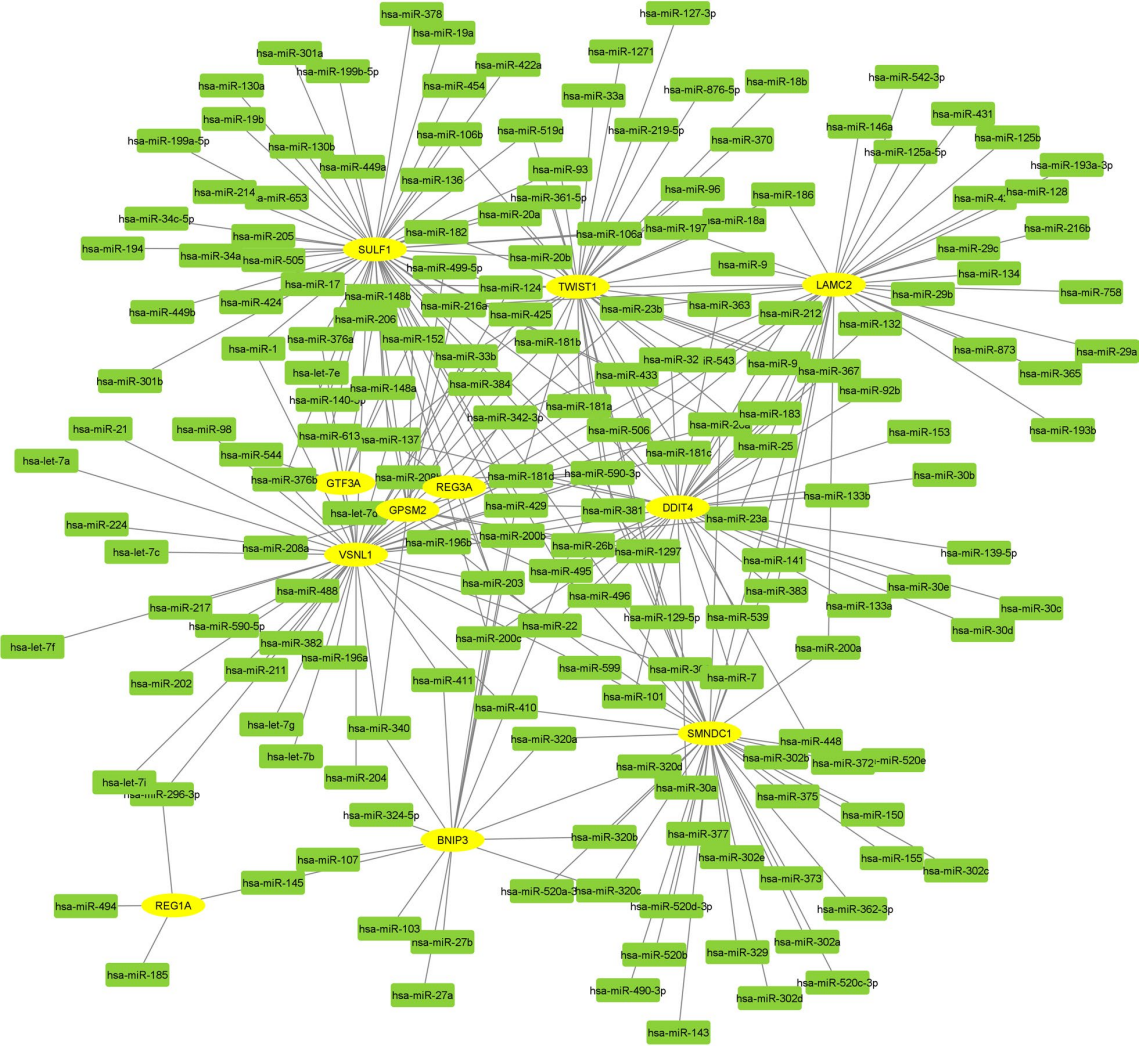
mRNA–miRNA bipartite network for selected genes in Cytoscape. Eleven key genes is yellow nodes and miRNAs have been indicated in green color that were predicted from Target scan, DIANA, miR2Disease, miRWalk, miRNet, and microRNA.org

One mRNA-miRNA bipartite network was created from key genes and miRNAs related to them (Fig. 4 and Additional file 3: Table S2). Nine miRNAs with the highest degree and most common for 11 key genes (hub miRNAs) were selected as most effective miRNAs on the network of CRC (hsa-miR-1, hsa-miR-125a-5p, hsa-miR-129-5p, hsa-miR-1297, hsa-miR-137, hsa-miR-145, hsa-miR-148b, hsa-miR-181d and hsa-miR-185). To reduce analysis complexity, lncRNAs for hub miRNAs were predicted using miRNA**–**lncRNA target algorithms in several databases (Additional file 4: Table S3). The Topmost of lncRNAs (18 lncRNAs), those experimentally have been supported are summarized in Table 2.

**Table 2:** Topmost of predicted lncRNAs based on 9 hub miRNAs in colorectal cancer

lncRNAs		
LINC01206	MEG3	PAX8-AS1
HCG18	MUC19	TTN-AS1
TPT1-AS1	SNHG14	RPL34-AS1
SATB1-AS1	ZNRD1-AS1	AC009264.1
TPTEP1	TCL6	ENTPD1-AS1
LMCD1-AS1	FAM95B1	SLC8A1-AS1

**Fig. 5:**
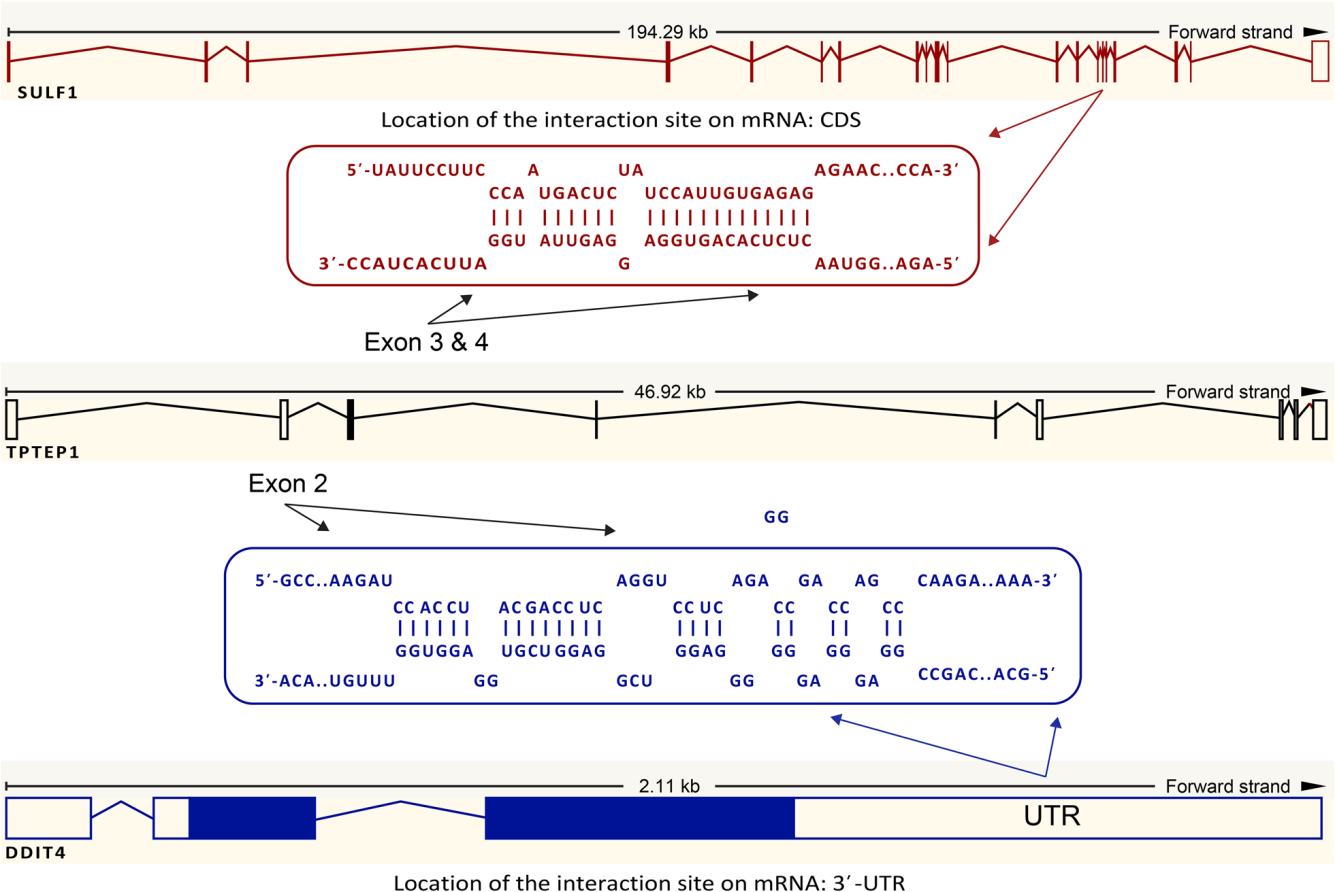
mRNA–lncRNA interactions (DDIT4 and SULF1 with TPTEP1). DDIT4 and SULF1 have interaction sites for TPTEP1, the links predicted in interactions DDIT4 and SULF1 with TPTEP1 are in untranslated region (UTR) and coding sequence (CDS) region, respectively

Finally, reviewing the literature led to selecting genes of DDIT4, SULF1, miR-181d-5p and miR-148b-3p that are involved in CSCs amongst the key genes and hub miRNAs for our experimental validation [68–71]. Also, TPTEP1 was selected on topmost of the predicted lncRNAs for our experimental study that has target and interaction sites in untranslated region (UTR) and coding sequence (CDS) region for DDIT4 and SULF1, respectively, as found by prediction algorithms (Fig. 5).

### Experimental studies in CRC tissues and colorectal CSC-enriched spheroids

#### Patients’ characteristics

Out of 48 patients with CRC, the number of males and females were 29 (60.4%) and 19 (39.6%) respectively. The patients were in the age group of 20–87 years with the mean age of 59 ± 13.7 (mean ± SD) years. Twenty-five (52.1%) of the cases were in early stages (I-II) while 23 (47.9%) were in advanced stages (III-IV) of the tumor. Twenty-one patients (43.8%) had well differentiated cancer cells (Grade 1), followed by 19 cases (39.6%) with moderately differentiated (Grade 2) and 8 cases (16.6%) with poorly differentiated cancer cells (Grade 3). Patients were classified into 3 groups: 25 patients (52.1%) didn’t have any metastasis, 14 patients (29.1%) had only lymph node metastasis and 9 patients (18.7%) had distant metastasis. Vascular and perineural invasion were observed in 14 (29.1%) and 18 (37.5%) patients respectively.

#### RNA expression levels of selected genes in CRC tissues and the relationship with clinicopathological features

**Fig. 6:**
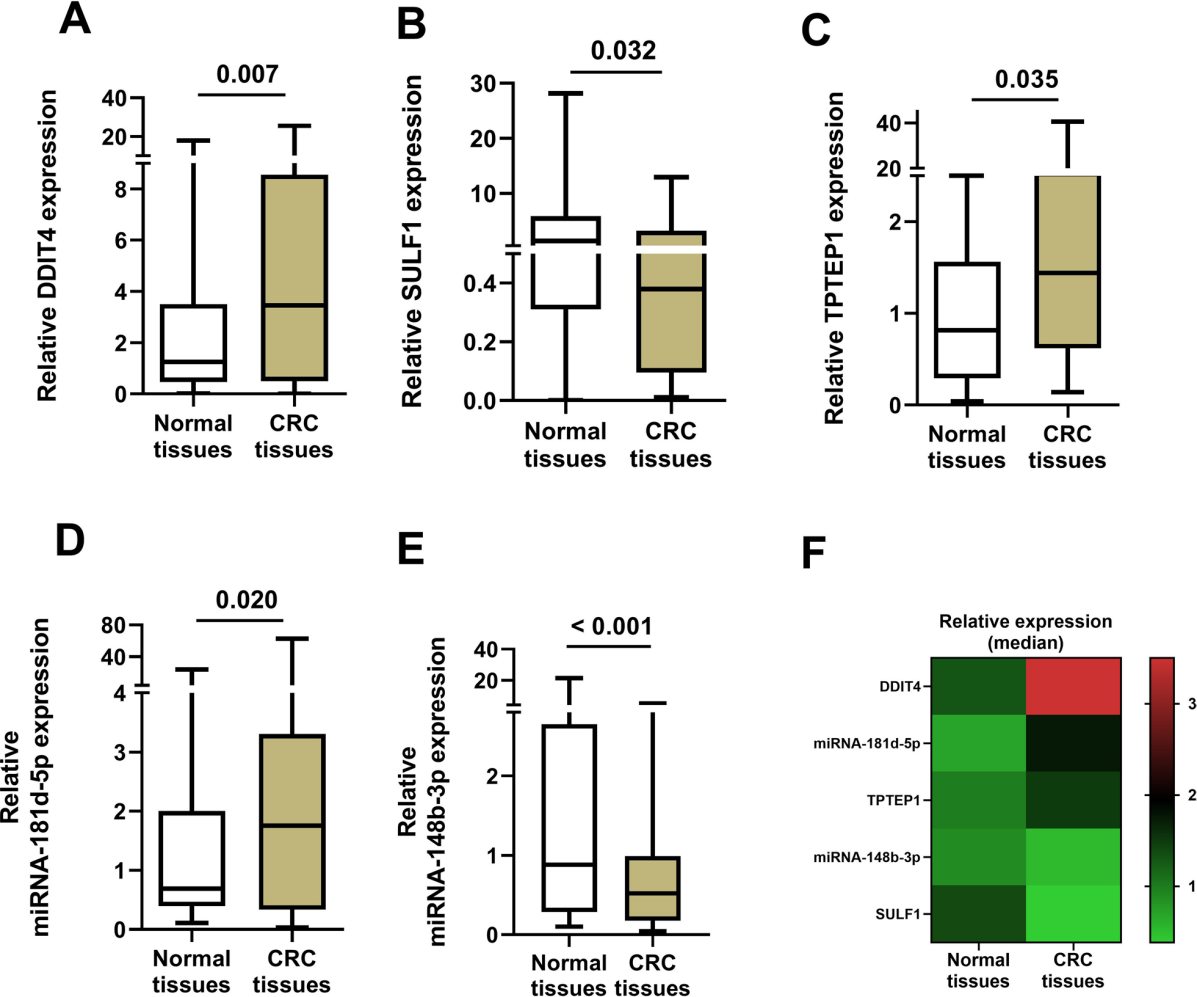
Boxplot of RT-qPCR data presented as median (min–max) for comparing expression levels of genes between colorectal cancer (CRC) tissues and adjacent normal tissues. Expression levels of DDIT4 (**A**), SULF1 (**B**), TPTEP1 (**C**), miR-181d-5p (**D**), miR-148b-3p (**E**), and summary of the frame A-E as a simple heat map (**F**) from CRC tissues compared to adjacent normal tissues (control)

The mRNAs and miRNAs expression levels were evaluated in 48 tumor tissues and their adjacent normal tissues of CRC patients by RT-qPCR. The analysis of the RT-qPCR data using Mann–Whitney *U* test demonstrated median expression levels of DDIT4 (*P* = *0.007*), TPTEP1 (*P* = *0.035*) and miR-181d-5p (*P* = *0.020*) were significantly higher in CRC tissues compared to the adjacent normal tissues (Table 3) (frame A, C and D of Fig. 6, respectively). In contrast, the expression levels of SULF1 (*P* = *0.032*) and miRNA-148b-3p (*P* < *0. 001*) were significantly lower in CRC tissues compared to the adjacent normal tissues (Table 3) (frame B and E of Fig. 6, respectively). Figure 6F summaries the data of frame A-E in a heat map graph showing the pattern of these gene expression from the up to down-regulated expression levels in CRC tissues compared to the adjacent normal tissues in order, where the DDIT4 shows the highest up-regulated and SULF1 the lowest down-regulated expression levels in CRC tissues compared to the adjacent normal tissues.

**Table 3:** Median expression of genes in tumor tissues compared to adjacent normal colorectal tissues

Genes	Normal	Tumor	Pattern	*p-*value
	Median 2^−ΔΔCt^	Median 2^−ΔΔCt^		
DDIT4	1.2978	3.5836	Up-regulated	0.007
SULF1	1.3811	0.3854	Down-regulated	0.032
TPTEP1	0.8150	1.4401	Up-regulated	0.035
miR-181d-5p	0.6961	1.8826	Up-regulated	0.020
miR-148b-3p	0.8800	0.5170	Down-regulated	< 0.001

**Fig. 7:**
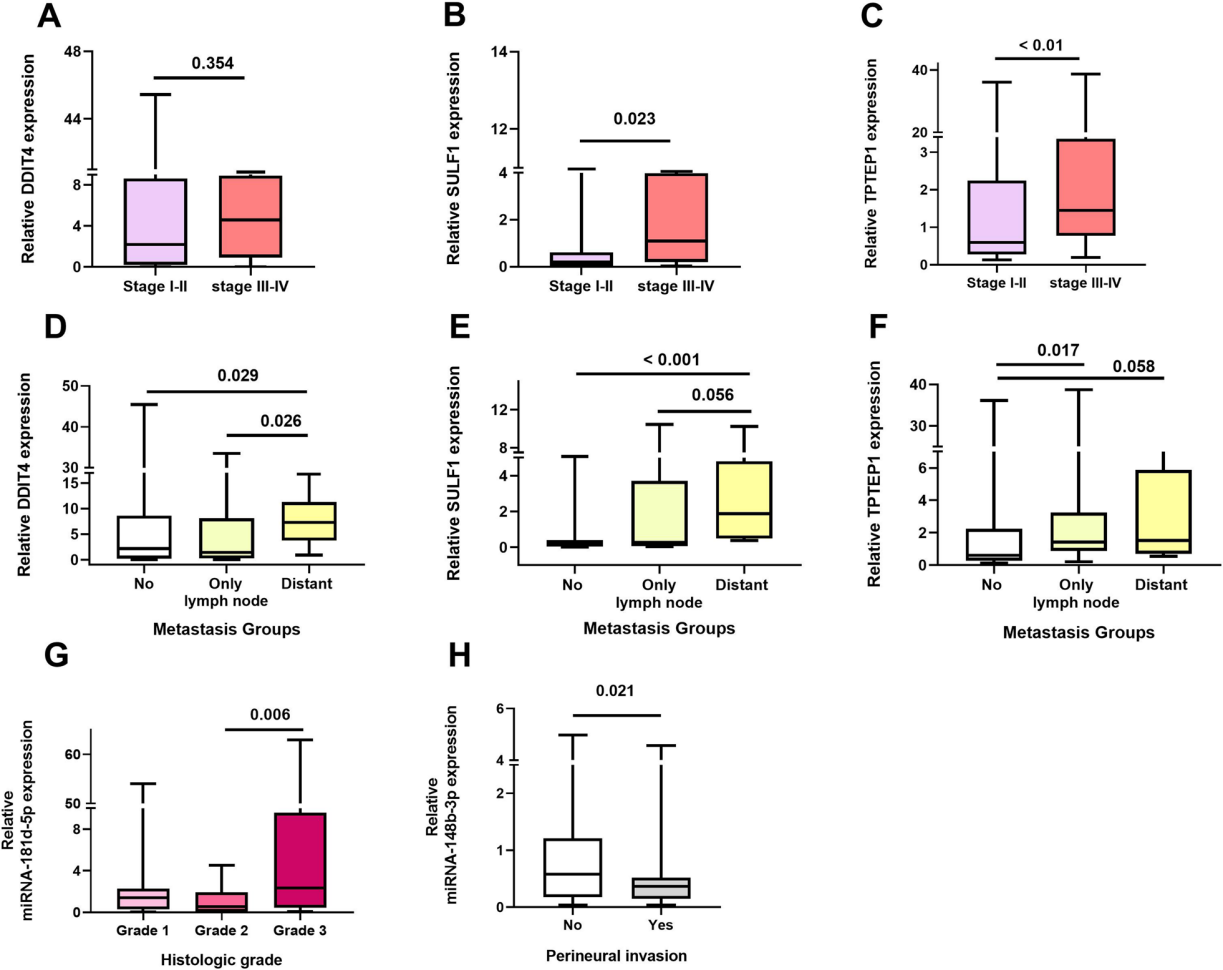
Relationship between expression levels of genes with clinicopathological features in tumor tissues from colorectal cancer (CRC) patients. Relationship expression of DDIT4, SULF1 and TPTEP1 with TNM stages; statistical significantly expression levels of SULF1 and TPTEP1 increased with advanced stage (**A–C**). Increased expression levels of DDIT4, SULF1, and TPTEP1 were observed in CRC patients with metastasis (**D–F**). Higher expression level of miR-181d-5p was associated with histologic grade in CRC tissues (**G**). miR-148b-3p expression reduced in CRC patients with perineural invasion (**H**)

Additional analyses were performed to find any association between the median expression of the selected genes and the clinicopathological features of the CRC patients (Table 4). Results displayed significant relationship between TNM stage and expression of SULF1 (*P* = *0.023*) and TPTEP1 (*P* < *0.01*) in CRC patients. Median expression of SULF1 and TPTEP1 showed significantly increased levels in tumor tissues obtained from CRC patients with the advanced stages (frame B and C of Fig. 7). Kruskal–Wallis test showed that expression levels of DDIT4 (*P* = *0.048*), SULF1 (*P* = *0.009*) and TPTEP1 (*P* = *0.035*) were significantly related to metastasis (Table 4). Our results demonstrated median expression of DDIT4 (*P* = *0.029*) and SULF1 (*P* < *0.001*) were significantly higher in patients with distant metastasis than patients without metastasis (frame D and E of Fig. 7) while median expression of TPTEP1 was higher in patients with only lymph node metastasis than patients without metastasis (*P* = *0.017*) (frame F of Fig. 7). The levels of miR-181d-5p expression were found significantly related to the histologic grading of the CRC tumor (*P* = *0.016*) (Table 4). As shown in frame G of Fig. 7, the median expression levels of miR-181d-5p was significantly higher in CRC patients with Grade 3 than patients with Grade 2 (*P* = *0.006*). A significant relationship between median expression levels of miR-148b-3p and presence of perineural invasion was observed, where the median expression levels of miR-148b-3p in patients with the present of perineural invasion were significantly lower compared to that in patients without the perineural invasion (*P* = *0.021*) (Table 4 and frame H of Fig. 7).

**Table 4:** Relationship between RNA expression of genes and clinicopathological features from colorectal cancer samples

Clinicopathological features	Relevant expression of genes (2 ^−ΔΔCt^), *p-*value				
	DDIT4	SULF1	TPTEP1	miR-181d-5p	miR-148b-3p
Tumor size (cm) , mean ≈ 5 cm					
$\le$ 5	0.689	0.468	0.076	0.430	0.556
$>$ 5					
Vascular invasion					
Yes	0.273	0.133	0.193	0.280	0.315
No					
Perineural invasion					
Yes	0.752	0.549	0.966	0.058	0.021*
No					
TNM stage					
I-II	0.354	0.023*	< 0.01*	0.920	0.359
III-IV					
Metastasis					
No metastasis	0.048*	0.009*	0.035*	0.681	0.226
Only lymph node metastasis					
Distant metastasis					
Histologic grade (Tumor differentiation)					
Grade 1 (Low)	0.922	0.215	0.978	0.016*	0.062
Grade 2 (Moderately)					
Grade 3 (Poorly)					

^*^ Values in bold are statistically significant (P < 0.05)

**Fig. 8:**
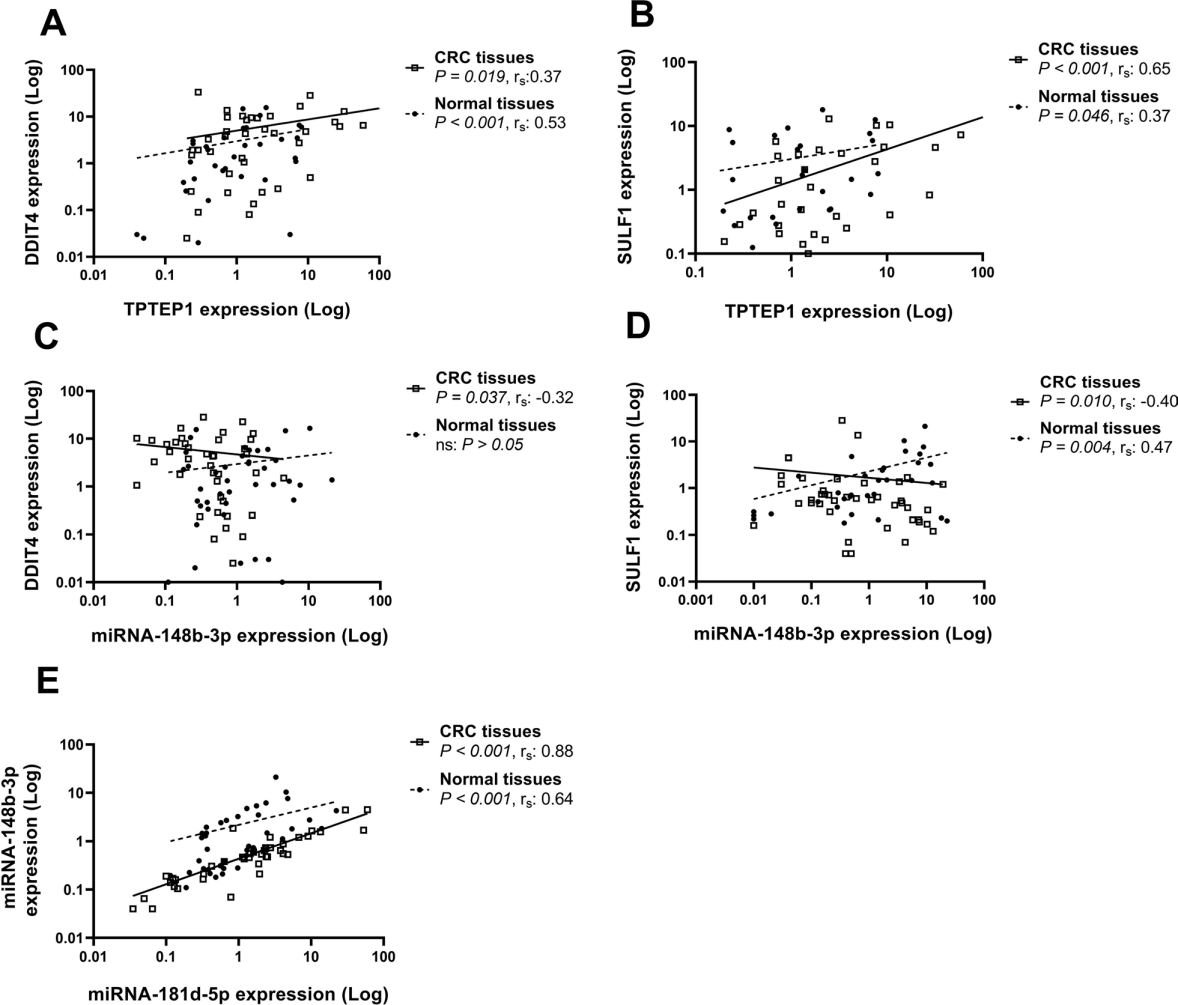
Significant correlations for expression levels between DDIT4 and SULF1 with ncRNAs (TPTEP1 and miR-148b-3p). Correlations between DDIT4 and TPTEP1 (**A**), SULF1 and TPTEP1 (**B**), DDIT4 and miR-148b-3p (**C**), SULF1 and miR-148b-3p (**D**) and miR-148b-3p and miRNA-181d-5p (**E**) in colorectal cancer (CRC) tissues and adjacent normal tissues (control)

There were numerous significant correlations between the RNA expression levels of the selected genes amongst each other both in CRC tissues (Table 5) and adjacent normal tissues (Table 6) as shown by spearman correlation values. The expression levels of TPTEP1 were positively correlated with the expression levels of DDIT4 both in CRC (*P* = *0.019*, r_s_: 0.37) and adjacent normal tissues (*P* < *0.001*, r_s_: 0.53) (frame A of Fig. 8). Also, a significant positive correlation was found between TPTEP1 and SULF1 expression levels both in CRC tissues (*P* < *0.001*, r_s_: 0.65) and to a lesser extent in adjacent normal tissues (*P* = *0.046*, r_s_: 0.37) (frame B of Fig. 8). There was a negative correlation between DDIT4 and miR-148b-3p expression levels in CRC tissues (*P* = *0.037*, r_s_: − 0.32) (frame C of Fig. 8). Moreover, a significant negative correlation between SULF1 and miR-148b-3p expression levels in CRC tissues (*P* = *0.010*, r_s_: − 0.40) and to a greater extent, but positively, in adjacent normal tissues (*P* = *0.004*, r_s_: 0.47) was found (frame D of Fig. 8). The expression levels of miR-148b-3p were positively and strongly correlated with the expression levels of miRNA-181d-5p both in CRC (*P* < *0.001*, r_s_: 0.88) and adjacent normal tissues (*P* < *0.001*, r_s_: 0.64) (frame E of Fig. 8).

**Table 5:** Correlation coefficients according to Spearman between RNA expression of genes for all tumor tissues from colorectal cancer patients

	TPTEP1	miR-181d-5p	miR-148b-3p
DDIT4	0.37*	− 0.25	− 0.32*
SULF1	0.65**	− 0.23	− 0.40*
TPTEP1	1.00	− 0.007	− 0.20
miR-181d-5p		1.00	0.88**
miR-148b-3p			1.00

^**^ Correlation is significant at the 0.01 level

^*^ Correlation is significant at the 0.05 level

**Table 6:** Correlation coefficients according to Spearman between RNA expression of genes for all adjacent normal tissues from colorectal cancer patients

	TPTEP1	miR-181d-5p	miR-148b-3p
DDIT4	0.53**	− 0.29	0.16
SULF1	0.37*	− 0.11	0.47**
TPTEP1	1.00	− 0.10	0.25
miR-181d-5p		1.00	0.64**
miR-148b-3p			1.00

^**^Correlation is significant at the 0.01 level

^*^Correlation is significant at the 0.05 level

#### Validation of CSC marker genes and expression levels of selected genes in CSC-enriched spheroids compared to HT-29 cancer cells

**Fig. 9:**
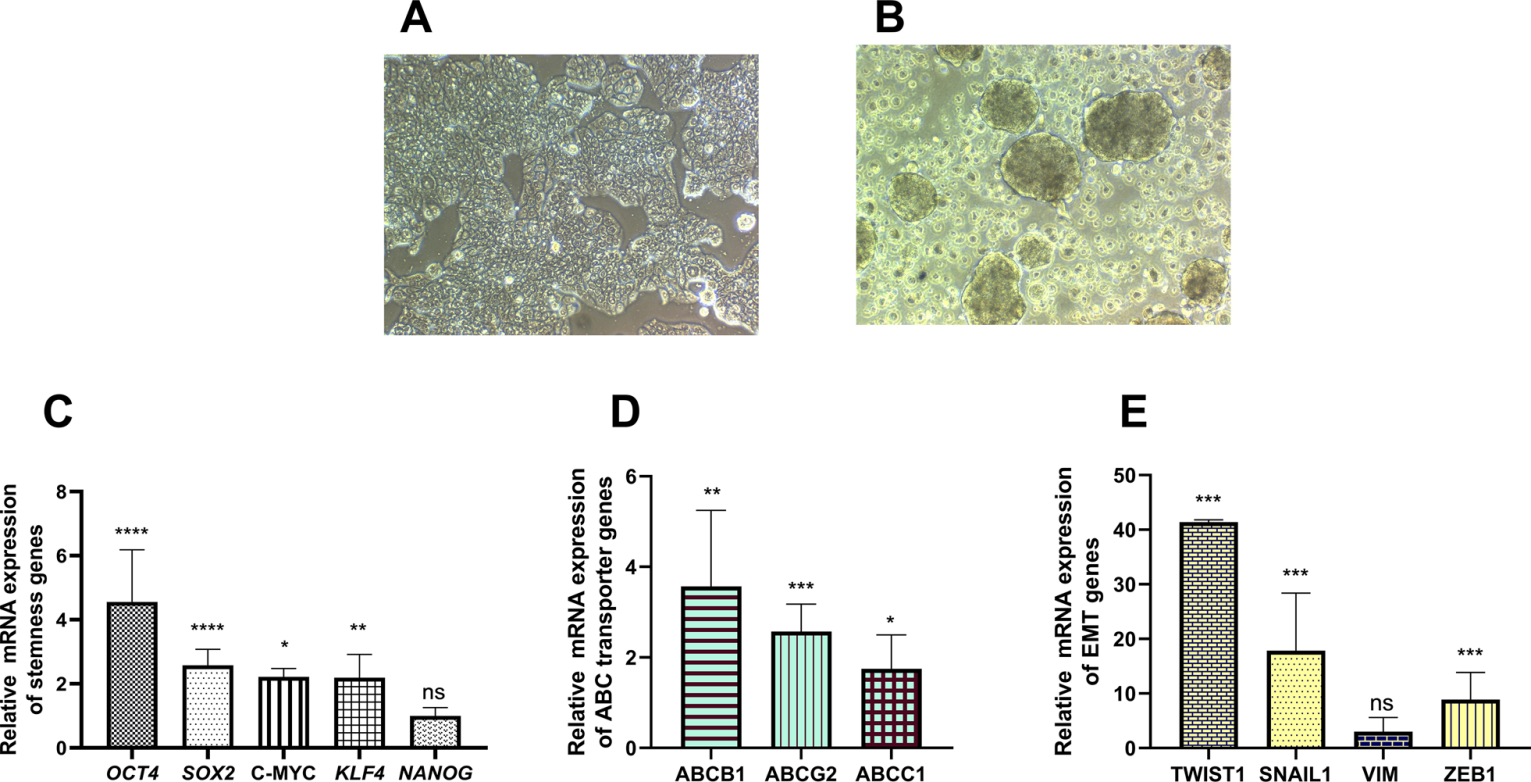
Evaluation cancer stem cell (CSC) features in colorectal CSC-enriched spheroids. Morphological feature of HT-29 cancer cells (**A**) and colorectal CSC-enriched spheroids derived from HT-29 cells (**B**). Overexpression of stemness genes (OCT4, SOX2, C-MYC, KLF4, and NANOG), (**C**), ABC transporter genes (ABCB1, ABCG2, and ABCC1) (**D**) and epithelial-mesenchymal transition (EMT) genes (TWIST1, SNAIL1, Vimentin (VIM), and ZEB1) (**E**) in colorectal CSC-enriched spheroids compared to HT-29 cancer cells as control. Not significant (ns): *P* > *0.05*, *P ≤ *0.05*, **P ≤ *0.01*, ***P ≤ *0.001*, ****P ≤ *0.0001*

We evaluated stemness, ABC transporter, and EMT marker genes as CSC features in CSC-enriched spheroids derived from HT-29 as determined by RT-qPCR after observation of sphere morphology under the microscope (frame A and B of Fig. 9). Our results showed significantly higher expression levels of stemness genes (OCT4, SOX2, C-MYC, and KLF4), ABC transporter genes (ABCB1, ABCG2, and ABCC1) and EMT genes (TWIST1, SNAIL1, and ZEB1) in colorectal CSC-enriched spheroids compared to HT-29 cancer cells (control) (frame C-E of Fig. 9).

**Fig. 10:**
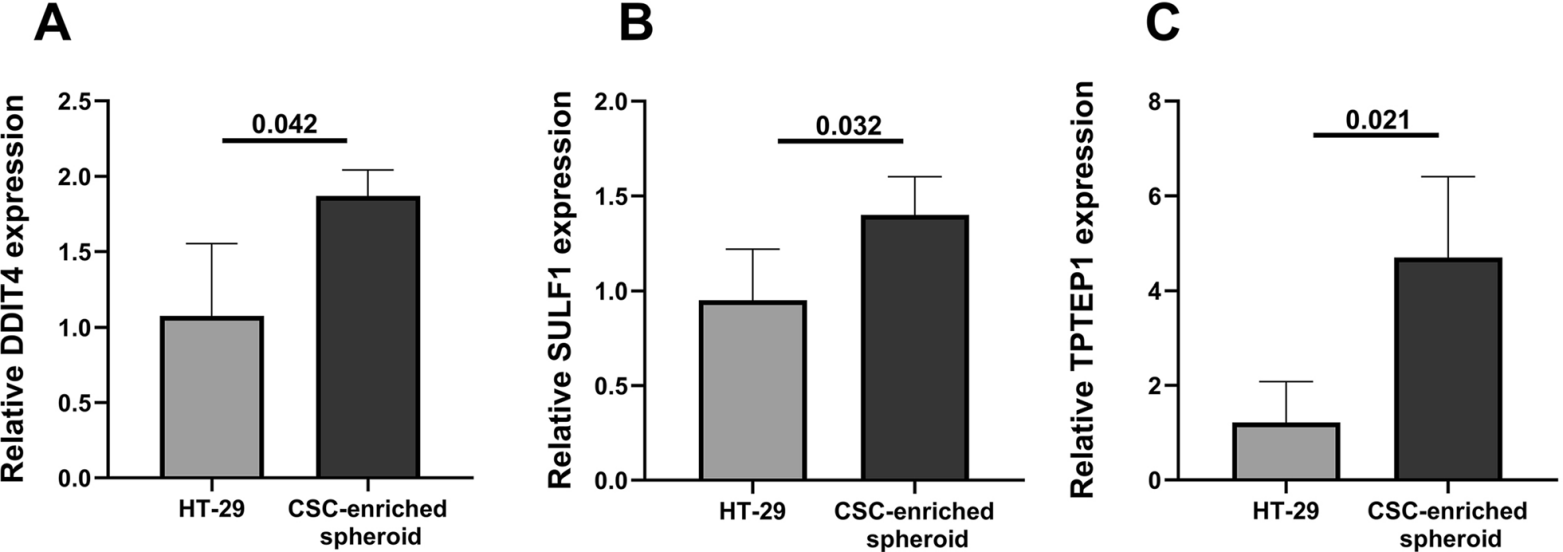
Expression levels of DDIT4, SULF1 and TPTEP1 in colorectal CSC-enriched spheroids. Higher expression of DDIT4, SULF1, and TPTEP1 was observed in colorectal CSC-enriched spheroids than HT-29 cancer cells which all were statistically significant. DDIT4 (**A**), SULF1 (**B**), and TPTEP1 (**C**)

After detection of CSC features, the RNA expression levels of DDIT4, SULF1, TPTEP1 and miRNAs (miR-181d-5p and miR-148b-3p) were measured using RT‐qPCR in colorectal CSC-enriched spheroids and HT-29 cancer cells. RNA expression levels of DDIT4 (*P* = *0.042*), SULF1 (*P* = *0.032*) and TPTEP1 (*P* = *0.021*) were significantly higher in colorectal CSC-enriched spheroids compared to the HT-29 cancer cells (frame A-C of Fig. 10). No significant difference was found in miRNAs expression levels (miR-181d-5p and miR-148b-3p) between colorectal CSC-enriched spheroids and HT-29 cancer cells.

## Discussion

Numerous RNAs (mRNAs, lncRNAs and miRNAs) can be used as potential biomarkers for diagnosis, prognosis and treatment in various cancers and their dysregulation shown to be associated with the development of different cancers. RNA biomarkers provide dynamics insights into cell regulation and processes compared to DNA biomarkers. They have more sensitivity and specificity than protein biomarkers [72]. The biological roles of RNAs make them as important as the functions of proteins [73]. The use of RNA studies in medicine has led to attracting numerous companies to develop new RNA-based diagnostic, prognostic tools, and drugs [74]. The panels such as ThyraMIR/ThyGENX and approval of the first RNAi drug Onpattro have made the RNAs, especially ncRNAs, studies important [74, 75].

In the current study, to identify RNA biomarkers in CRC, bioinformatics analysis was applied to detect DEGs in CRC microarray data, in which 11 key genes were selected for further analysis for predictions of miRNAs and lncRNAs. Then, we evaluated the expression levels of DDIT4, SULF1, TPTEP1, miR-181d-5p and miR-148b-3p, as potential biomarkers, in CRC patients and their association with clinicopathological features.

DDIT4 is a suppressor for mammalian target of rapamycin (mTOR) signaling pathway which is induced in various cellular stress conditions such as hypoxia and DNA damage [28, 76, 77]. Moreover, DDIT4 gene has a p53 transcription-factor binding site which can play a key role in the p53-dependent tumorigenesis [78]. Despite its repressive role on mTOR signaling pathway, up-regulation of DDIT4 has been shown to promote cell proliferation and reduce apoptotic rate in various cell types [79, 80]. DDIT4 was suitable candidate gene in order to predict miRNAs in CRC because of not only its participation in the PI3K/Akt/mTOR signaling pathway [28] but also its involvement in “miRNAs in cancer” pathway as shown by KEGG pathway analysis. We found RNA expression levels of DDIT4 were significantly higher in CRC tissues compared to the adjacent normal tissues. This result is in line with the study on gastric cancer displaying up-regulation of DDIT4 expression in the tumor tissues compared to the adjacent normal tissues as found by RT-qPCR and immunohistochemically staining [79]. Since earlier findings reported that inhibition of mTOR pathway is leading to enrichment of cancer stem cells, high DDIT4 expression could be related to expression of stem-cells markers [68, 81]. As expected, up-regulation of DDIT4 expression was observed in colorectal CSC-enriched spheroids compared to HT-29 cancer cells in our study. This result supports higher expression levels of DDIT4 in the CRC patients with metastasis because CSCs play role in tumor metastasis [82], and are key drivers in tumor progression [83]. These findings indicate that this higher RNA expression of DDIT4 is significantly associated with more aggressive tumor behavior. Our report is the first study to show high mRNA levels of DDIT4 expression and its clinical significance in CRC tissues as well as in colorectal CSC-enriched spheroids.

SULF1, another candidate gene, is a subtype of proteinase released by various cells in the extra cell matrix (ECM) and alters its function by modifying HS. This alteration affects several signaling molecules toward the development and spread of cancer in the microenvironment [84]. Several experimental studies reported SULF1 as a tumor suppressor effector and its down-regulation levels related to several cancers such as pancreatic, ovarian and gastric cancer [85–87]. While, some other studies have shown up-regulation of SULF1 expression in gastric, colorectal and bladder cancer [31, 88, 89]. Our bioinformatics analysis showed up-regulation of SULF1 expression levels. This data is consistent with the previous results on ONCOMINE database showing increased expression levels of SULF1 in CRC tissues compared to the adjacent normal tissues [90]. In our study, RT-qPCR data showed down-regulation of SULF1 expression levels in CRC tissues compared to the adjacent normal tissues, although its expression levels showed significantly increased levels in patients with more advanced stage and metastasis of the tumor. This result is in line with the previous findings observing down-regulating of SULF1 in early stage of ovarian tumors [86, 91]. The increased SULF1 expression levels have been also reported at the later stages of malignancy progression in CRC patients [32, 33]. It has been described that SULF1 has ambivalent functions and there is insufficient information to understand the conflicting results regarding the role of SULF1 in cancer [92, 93]. The tumor suppressor effect of SULF1 was described under hypoxic conditions in solid tumors. The level reduction of SULF1 in such environments causes increasing in 6-O-sulfate on HSPGs which subsequently leads to increasing of the fibroblast growth factor (FGF) signaling and cancer progression [91]. Besides, the oncogenic effect of SULF1 was proposed due to the high-affinity of HS-Wnt complex. In fact, extracellular removal of the 6-O-sulfate on the HSPGs by SULF1 allows initiation of the Wnt signaling [94, 95]. Evidence suggests that overexpression of SULF1 is related to expression of EMT genes and can promote EMT in human hepatocellular carcinoma [96]. In this regard, we measured SULF1 expression in colorectal CSC-enriched spheroids with increased EMT gene expression and observed significantly higher expression levels of SULF1 in colorectal CSC-enriched spheroids compared to the HT-29 cells. This finding is in line with the previous data showing up-regulation of SULF1 expression levels in high metastatic colorectal cancer cell lines [33] and breast CSCs [71]. Increased SULF1 expression levels in patients with distant metastasis, advanced stages of the tumor, as well as colorectal CSC-enriched spheroids indicate that the levels of SULF1 expression is being increased by tumor progression in CRC. Despite dysregulation of SULF1 expression levels in CRC, such challenging observations make it difficult to offer SULF1 as a “biomarker”. Therefore, more studies are needed to reveal the dual roles of SULF1 and its expression pattern in cancer patients.

In the present study, we also investigated expression of some ncRNAs including miRNAs (miR-181d-5p and miR-148b-3p) and TPTEP1 in CRC tissues. Previous reports indicated that miR-181d contributes in regulation of Akt pathway in breast cancer and CRC cell glycolysis which acts as an oncomiR [97, 98]. We demonstrated that miR-181d-5p is significantly up-regulated in tumor tissues compared with the adjacent normal tissues in CRC patients. This data is in good agreement with the previous data in CRC patients [97]. Moreover, the association between overexpression of miR-181d-5p and high-grade tumor cells may indicate a possible influence of increased miR-181d-5p expression in the progression of cancer. Despite what was previously described in the breast cancer cells and CRC patients about association between high expression of miR-181d-5p and increased invasion and migration of the tumors [97, 99], our data analysis didn't show any significant difference of miR-181d-5p expression levels with various metastasis groups in CRC patients. In line with this data, our results didn’t show any significant different in expression levels of miR-181d-5p between HT-29 cancer cells and colorectal CSC-enriched spheroids. While up-regulation of miR-181 family has been previously observed in the liver cancer stem/progenitor cells [70].

Cancer reports displayed that miR-148b, especially miR-148b-3p, plays an important role as a tumor suppressor by influencing on cell growth and proliferation [38], apoptosis [100], metastasis dissemination and cancer therapy responses [101]. Our result demonstrated a lower expression of miR-148b-3p in CRC tissues compared with the adjacent normal tissues which is in line with previous result in CRC patients [38]. Also, we observed down-regulation of miR-148b-3p in patients with vascular invasion compared to those without this invasion. No significant difference in miR-148b-3p expression was revealed between colorectal CSC-enriched spheroids and HT-29 cancer cells, nor between metastasis groups of CRC patients. In contrast to our findings, decreased expression and suppressor role of miR-148b-3p has been previously reported in the hepatic CSCs [69, 102].

Our investigation, for the first time, identified dysregulated expression of TPTEP1 in CRC patients. Contrary to the lung [40] and liver [41] cancer studies, the expression of TPTEP1 showed an up-regulation pattern in our CRC tissues compared to the adjacent normal tissues. Moreover, we observed higher expression of TPTEP1 in colorectal CSC-enriched spheroids than HT-29 cancer cells. As expected, based on predictions, DDIT4 and SULF1 expression levels were significantly correlated with the TPTEP1 expression levels in CRC. This result may be related to interaction between these RNAs amongst each other and can support findings about predicted binding sites based on bioinformatics algorithms for TPTEP1. According to the predicted binding site, DDIT4 in RNA level from 3′UTR region interacts with TPTEP1. It is remarkable that 3' UTRs play critical roles in gene expression regulation through bindings ncRNAs [103]. Also, RNA expression levels of DDIT4 and SULF1 were significantly correlated negatively with miR-148b-3p expression levels in CRC tissues. These correlations may be explained by regulatory effects of miR-148b-3p expression on these RNAs as predicted based on the mRNA-miRNA network. We aware that our research has limitations to describe in details these regulatory effects and further studies are warranted to understand the relationship between these RNAs.

## Conclusions

Overexpression of DDIT4 and TPTEP1 in CRC patients with metastasis and advanced stages as well as in colorectal CSC-enriched spheroids indicates that increased RNA expression of these markers may be useful indicators of more aggressive tumor behavior and further disease progression in CRC patients. Moreover, correlations and predicted interactions of TPTEP1 and miR-148b-3p with DDIT4 and SULF1 in mRNA level might be due to the regulatory effects of these RNAs amongst each other. According to the expression differences of DDIT4, SULF1, TPTEP1, miR-181d-5p, and miR-148b-3p in CRC tissues compared to the adjacent normal tissues, we believe our results provide a valuable resource in order to find biomarkers clinicopathologically relevant to CRC patients. From these findings, we are able to conclude that analysis of hub mRNA**–**miRNA genes can help to predict some important lncRNAs which are dysregulated in CRC patients.

## Supplementary Information

**Additional file 1: Table S1. ** Information obtained in the process of identification and selection of key genes including up-regulated genes with combined gene score > 3 of previous analysis (PMID: 31654507), up-regulated genes in carcinogenesis & colorectal cancer of DisGeNET with pvalue < 0.0001, the largest cluster of k-means clustering in STRING (confidence ≥ 0.4), and key genes.

**Additional file 2: Figure S1.** Protein–protein interaction (PPI) network analysis. PPI network explored the interactions between the 370 up-regulated genes with confidence ≥ 0.4. Five main clusters were obtained of the k-means algorithm that five colors were applied for indication gene clusters (each color indicates a cluster gene). **Figure S2.** Pathway analysis for the largest cluster covering 167 genes on Enrichr. Top ten results of pathway analysis that was performed based on BioPlanet, KEGG, WikiPathways, and Reactome libraries. **Figure S3.** Gene ontology analysis for the largest cluster covering 167 genes on Enrichr. Top ten results of gene ontology (GO) based on p-value. Results of GO analysis was contained cellular component (CC), biological process (BP), molecular function (MF) and Jensen diseases.

**Additional file 3: Table S2.** Information of miRNA-mRNA network for 11 key genes.

**Additional file 4: Table S3.** All of the predicted lncRNAs for 9 hub miRNAs.

## Data Availability

The analyzed data during the current study are available from the corresponding authors on reasonable request.

## Abbreviations

CRC: Colorectal cancer
CSCs: Cancer stem cells
ncRNAs: Non-coding RNAs
LncRNA: Long noncoding RNA
miRNA: MicroRNA
HS: Heparan sulfate
HSPGs: Heparan sulfate proteoglycans
DEGs: Differentially expressed genes
GO: Gene ontology
PPI: Protein–protein interaction
ECM: Extra cell matrix
EMT: Epithelial-mesenchymal transition
FGF: Fibroblast growth factors
