# Supplementary material for: Overexpression of DDIT4 and TPTEP1 are associated with metastasis and advanced stages in colorectal cancer patients: a study utilizing bioinformatics prediction and experimental validation
Source: Cancer Cell Int. 2021 Jun 9;21:303. doi: 10.1186/s12935-021-02002-x (PMC8191213; doi:10.1186/s12935-021-02002-x)
Supplement: Supplementary file 2 — Additional file 2: Figure S1. Protein–protein interaction (PPI) network analysis. PPI network explored the interactions between the 370 up-regulated genes with confidence ≥ 0.4. Five main clusters were obtained of the k-means algorithm that five colors were applied for indication gene clusters (each color indicates a cluster gene). Figure S2. Pathway analysis for the largest cluster covering 167 genes on Enrichr. Top ten results of pathway analysis that was performed based on BioPlanet, KEGG, WikiPathways, and Reactome libraries. Figure S3. Gene ontology analysis for the largest cluster covering 167 genes on Enrichr. Top ten results of gene ontology (GO) based on p-value. Results of GO analysis was contained cellular component (CC), biological process (BP), molecular function (MF) and Jensen diseases. [file 12935_2021_2002_MOESM2_ESM.docx]

**Overexpression of DDIT4 and TPTEP1 are associated with metastasis and advanced stages in colorectal cancer patients: a study utilizing bioinformatics prediction and experimental validation**

Fahimeh Fattahi, Jafar Kiani, Mahdi Alemrajabi, Ahmadreza Soroush, Marzieh Naseri, Mohammad Najafi^*^and [Zahra Madjd](https://www.ncbi.nlm.nih.gov/pubmed/?term=Madjd%20Z%5BAuthor%5D&cauthor=true&cauthor_uid=31576171)^*^

**Corresponding authors**

1- Professor. Zahra Madjd, M.D, Ph.D.

Oncopathology Research Center, Iran University of Medical Sciences, Tehran, Iran

[Zahra.madjd@yahoo.com](mailto:Zahra.madjd@yahoo.com), [majdjabari.z@iums.ac.ir](mailto:majdjabari.z@iums.ac.ir)

2- Dr. Mohammad Najafi, Ph.D.

Faculty of Medical Sciences, Iran University of Medical Sciences, Tehran, Iran

[nbsmmsbn@iums.ac.ir](mailto:nbsmmsbn@iums.ac.ir)


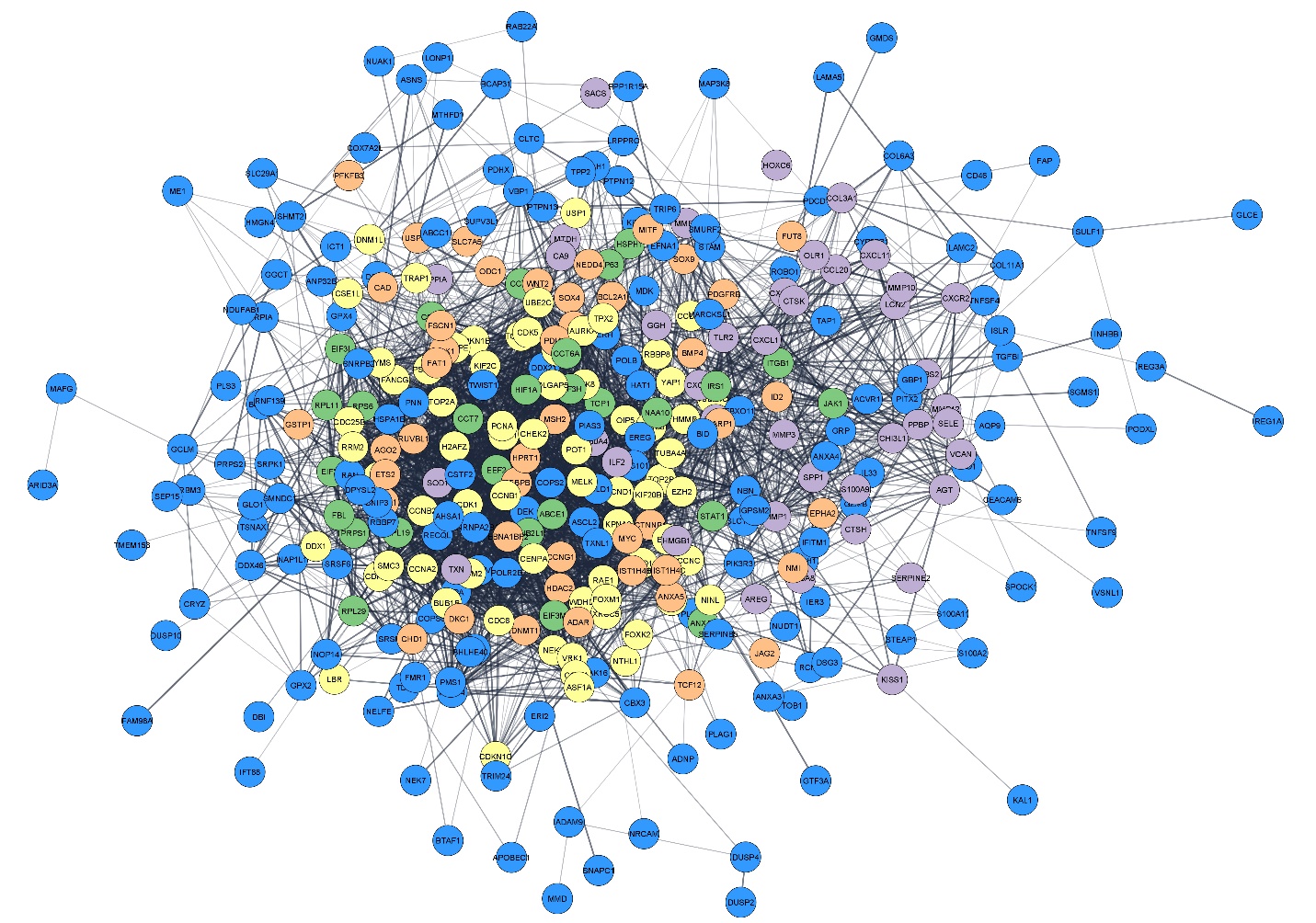


**Figure S1. Protein-protein interaction (PPI) network analysis.** PPI network explored the interactions between the 370 up-regulated genes with confidence ≥ 0.4. Five main clusters were obtained of the k-means algorithm that five colors were applied for indication gene clusters (each color indicates a cluster gene).


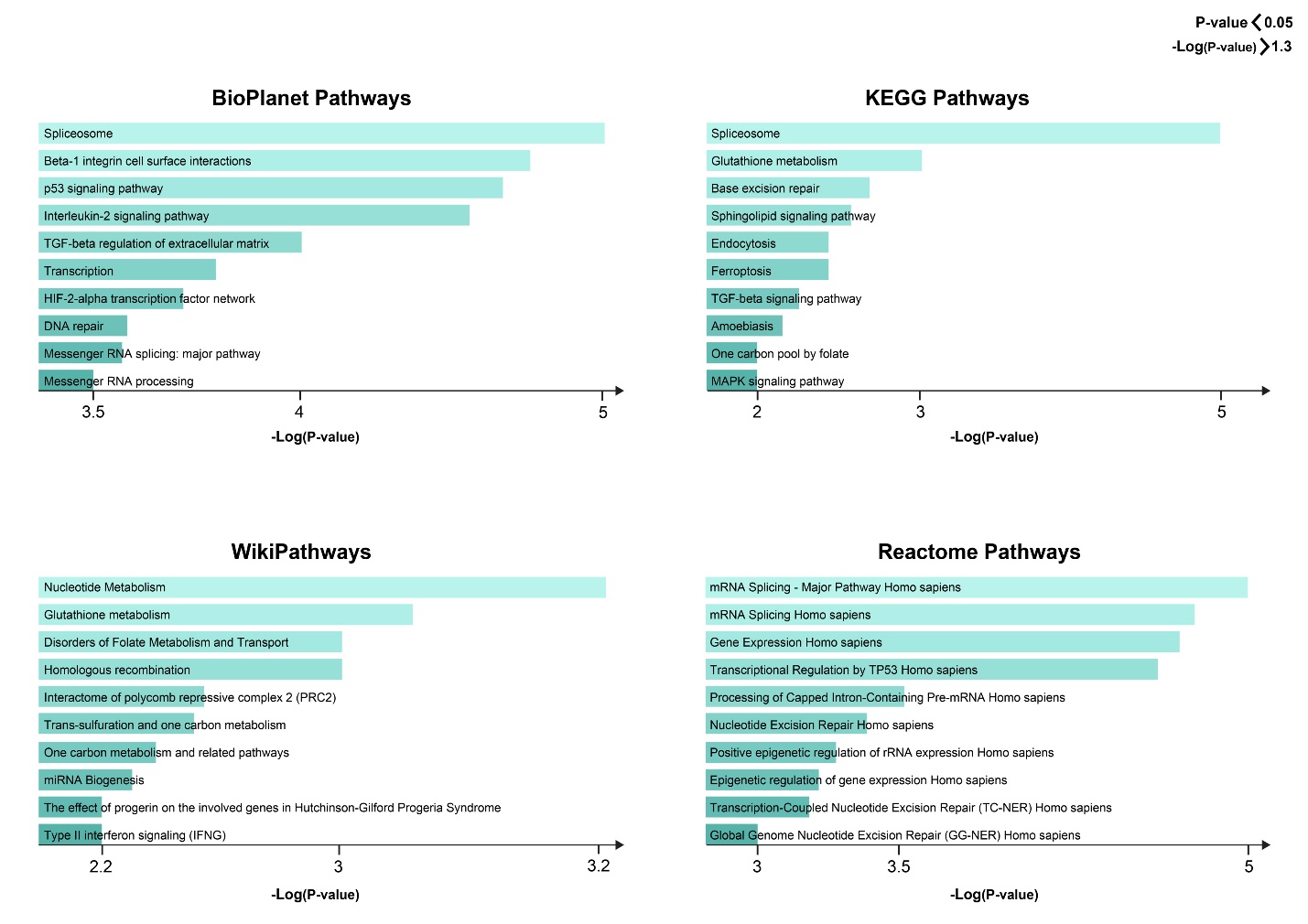


**Figure S2. Pathway analysis for the largest cluster covering 167 genes on Enrichr.** Top ten results of pathway analysis that was performed based on BioPlanet, KEGG, WikiPathways, and Reactome libraries.


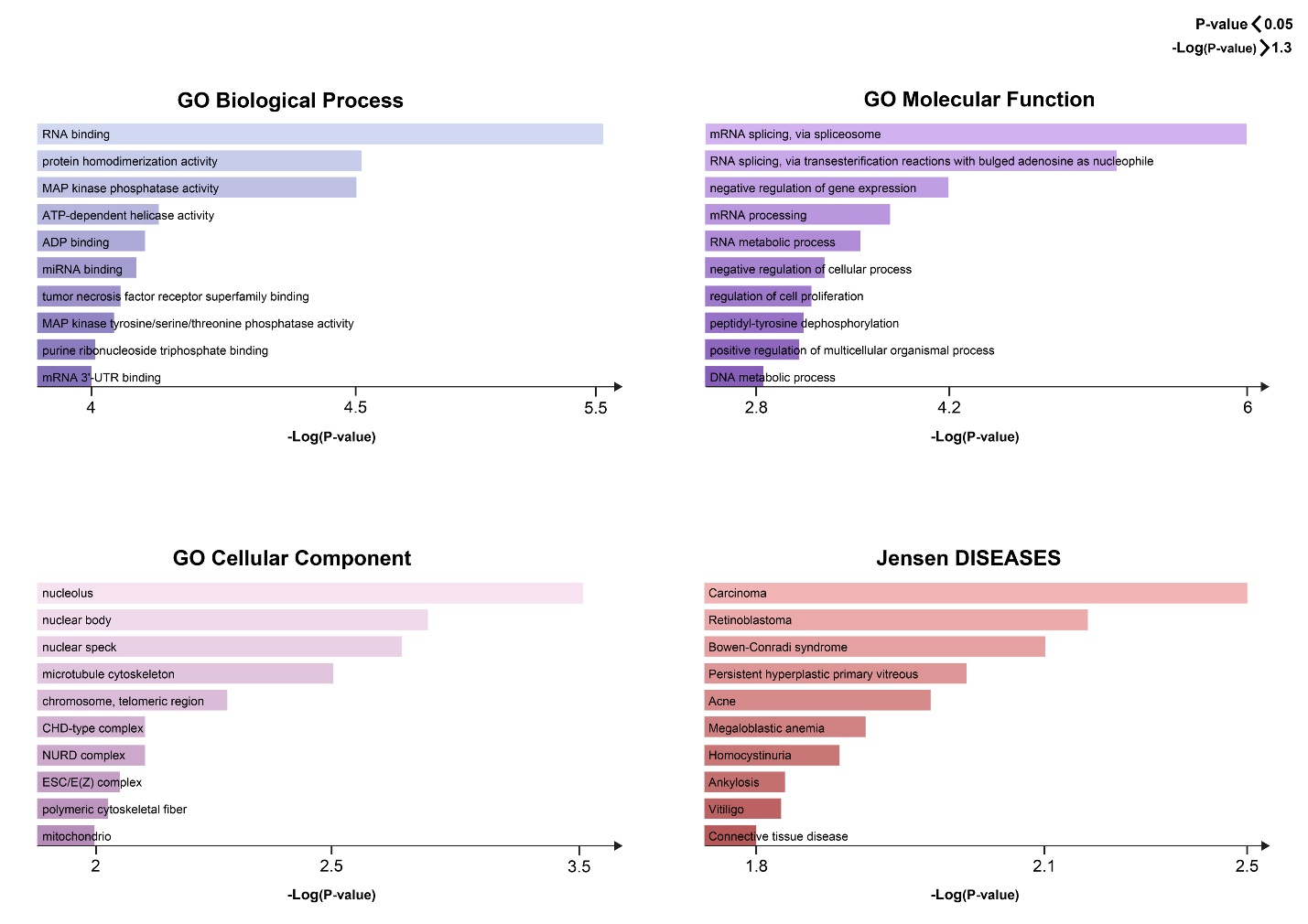


**Figure S3. Gene ontology analysis for the largest cluster covering 167 genes on Enrichr.** Top ten results of gene ontology (GO) based on p-value. Results of GO analysis was contained cellular component (CC), biological process (BP), molecular function (MF) and Jensen diseases.
